# Acupoint Application in Patients with Chronic Stable Angina Pectoris: Study Protocol of a Randomized, Double-Blind, Controlled Trial

**DOI:** 10.1155/2014/619706

**Published:** 2014-08-27

**Authors:** Yulan Ren, Dehua Li, Hui Zheng, Junling Lv, Junyan Leng, Linglin Zhang, Jie Zhang, Hailong Fan, Fanrong Liang

**Affiliations:** ^1^The Department of Acupuncture & Tuina, Chengdu University of Traditional Chinese Medicine, Chengdu, Sichuan 610075, China; ^2^The Department of Acupuncture, Teaching Hospital of Chengdu University of Traditional Chinese Medicine, Chengdu, Sichuan 610072, China

## Abstract

*Background*. Chronic stable angina pectoris (CSAP) is a major syndrome of ischemic heart disease (IHD). CSAP manifests as chest pain or discomfort and affects patients' quality of life. Acupoint application (AP) has been reported to be effective for managing the symptoms of CSAP, but the evidence is not convincing. Therefore, we designed a randomized, double-blind, placebo-controlled clinical trial to evaluate the efficacy of AP in the treatment of CSAP. *Methods and Analysis*. Two hundred participants with CSAP will be randomly assigned in a 1 : 1 : 1 : 1 ratio into 4 groups. All participants will receive 12 sessions of treatment in 4 weeks and the same basic treatment procedure. The participants will be visited and assessed for 12 weeks, including a 4-week screening, a 4-week treatment phase, and a 4-week follow-up phase. The primary outcome is the change in the total frequency of self-reported angina attack at 4th week compared with the baseline. The secondary outcomes include the intensity of angina pain, consumption of nitroglycerin or *Suxiao Jiuxin* pills, CCS angina classification, SAQ, SAS and SDS score. *Ethics*. The study protocol has been reviewed and approved by the Sichuan Regional Ethics Review Committee on TCM (number 2013kl-001). This trial is registered with clinicaltrials.gov NCT02029118.

## 1. Introduction

Ischemic heart disease (IHD) is the leading cause of death in high-income and in low- and middle-income countries and accounted for 17.3% and 11.8% of total deaths, respectively [1]. Chronic stable angina pectoris (CSAP) is the most prevalent manifestation of IHD and affects up to 5% of the over-40-year-old population in most developed countries. In every one million people in the general population of most European countries, it is estimated that 20,000 to 40,000 individuals suffer from CSAP [2, 3].

CSAP patients complain of severe chest pain or discomfort in the chest or adjacent areas. The pain and discomfort are caused by severe atherosclerotic narrowing of one or more coronary arteries. The current standard of care for CSAP relies on strategies that not only relieve symptoms and prolong angina-free walking but also reduce the incidence of adverse clinical outcomes. These strategies include lifestyle changes, aggressive management of modifiable coronary artery disease risk factors, pharmacological therapy, and myocardial revascularization [4, 5]. Despite improved diagnostic techniques, medical therapy, and revascularization procedures, many patients continue to suffer from angina [2, 5]. However, widely used antianginal therapies have side effects, including headache, postural hypotension and dizziness, and the continuous uptake of nitrates may lead to drug tolerance [6]. Therefore, complementary therapies are popular among CSAP patients [7, 8].

As one of the complementary therapies, acupoint application (AP) is an external therapy used in traditional Chinese medicine (TCM); with the aim of preventing and treating diseases, herbal medicines are made into plaster to be applied on certain acupoints. The treatment relies on the mechanism of percutaneous absorption, which is currently under study internationally for transdermal drug delivery [9]. AP is a straightforward procedure with few side effects. AP has a long history in China and is widely used as an alternative and complementary therapy for the treatment of stable angina. Prospective clinical studies have confirmed that AP may alleviate symptoms and improve heart function in patients with stable angina [10–12]. Experimental research has revealed that AP may alter electrical and mechanical cardiac activities, strengthen myocardial substrate metabolism in the margin of the ischemic region, and minimize myocardial ischemic injury [13]. However, the majority of published studies have been small-sample observational studies rather than intervention studies. A lack of high-quality randomized, controlled, trial results means that there is insufficient evidence for the use of AP as a therapy for stable angina. To provide more persuasive evidence, we designed a randomized, controlled, double-blind clinical trial to evaluate the efficacy and safety of AP in patients with CSAP.

## 2. Methods

### 2.1. Design

**Figure 1:**
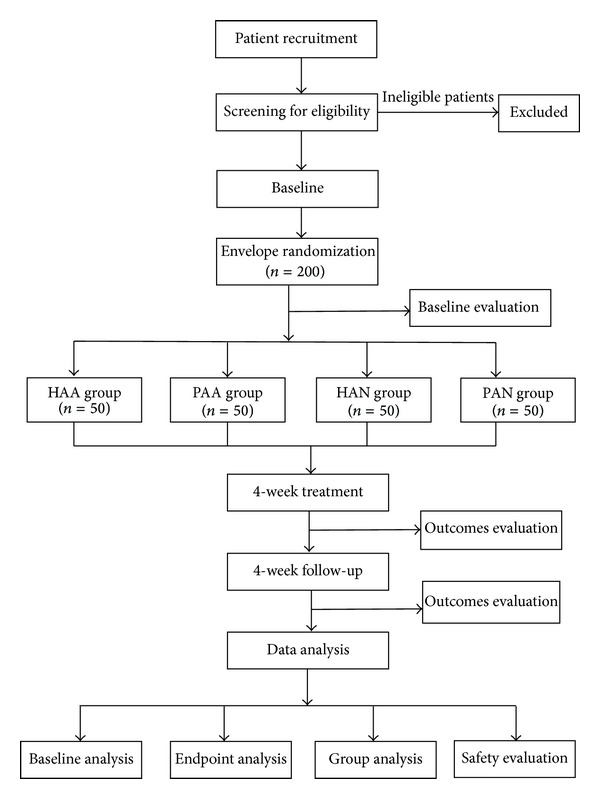
Study design.

This study is a randomized, double-blind, placebo-controlled trial that was designed to evaluate the efficacy and safety of AP in patients with CSAP in China. A total of 200 participants will be randomized (in a 1 : 1 : 1 : 1 ratio) into four groups: a herbal medicine application on acupoints (HAA) group, placebo application on acupoints (PAA) group, herbal medicine application on nonacupoints (HAN) group, and placebo application on nonacupoints (PAN) group (Figure 1). Eligible patients will be recruited from the Chengdu Chronic Disease Hospital, General Hospital of the Chengdu Military Region of the People's Liberation Army, and Third Affiliated Hospital of Chengdu University of Traditional Chinese Medicine.

The study protocol was approved by the Sichuan Regional Ethics Review Committee on TCM (number 2013kl-001) in January 2013. The study protocol follows the principles of the CONSORT and STRICTA statements as well as the Declaration of Helsinki (Sixth revision, 2008). Each patient will sign an informed consent form prior to any study procedure being conducted. Meanwhile, the patients will have sufficient time to decide whether they will participate in this study or select other treatments.

### 2.2. Study Flow

When a patient with CSAP visits a doctor in the cardiovascular department of one of the three study centers, the doctor will provide information about the trial and ask if the patient would like to participate in the study.

First, the patient will be informed. After enrollment, the patient will receive basic treatment combined with AP with herbal medicine/placebo plaster on acupoints/nonacupoints.

During the observation period of 12 weeks, patients are not permitted to receive any other treatments except drugs for immediate relief of acute symptoms of CSAP.

If a patient consents, the investigator will ask questions to satisfy the inclusion and exclusion criteria. The investigator will also collect demographic data and information about medical history, concomitant medication, physical examination, vital signs, and regular test results. If the patient is eligible, he or she will be randomly assigned to one of the four groups based on a sequence sealed in an opaque envelope. The following parameters will be evaluated at baseline: the frequency of angina attack, consumption of nitroglycerin or* Suxiao Jiuxin* pills, visual analogue scale (VAS) score, Canadian Cardiovascular Society (CCS) angina classification, Seattle Angina Questionnaire (SAQ) score, self-rating anxiety scale (SAS) score, and self-rating depression scale (SDS) score. Efficacy and safety will be evaluated at the 4th week and 8th week.

Each patient will be required to complete a participant diary to record symptoms, medications, and adverse events (AEs); the diaries will be given to the investigator at every visit.

### 2.3. Randomization and Blinding

Complete randomization will be stratified for centers in this study. Using the method of complete randomization, the randomization codes will be generated by Statistical Analysis Software (SAS, 9.1). Randomization codes will be managed by a statistician who will not be involved in the study. The plaster will be prepared beforehand by the central pharmacy and will be marked with labels and randomization codes. To ensure that the assigned intervention is concealed, the investigators will obtain an opaque, sealed envelope containing each participant's assigned intervention from the statistician just before the procedure is performed. The sealed envelope will be opened when an eligible patient is enrolled in the study. Then, the participant will be treated according to the allocation specified in the envelope. The investigators, participants, efficacy evaluators, and statisticians will all be blinded to the intervention allocations.

### 2.4. Participants

#### 2.4.1. Inclusion Criteria

Patients will be diagnosed according to the CSAP criteria that are issued by the American College of Cardiology/American Heart Association (ACC/AHA). Eligible participants are male or female, are aged between 35 and 85 years, have at least a 3-month history of stable angina, have at least 2 angina episodes per week, and agree to provide written informed consent.

#### 2.4.2. Exclusion Criteria

Patients will be excluded if they have a history of diabetes, coinfection, blood disorders, allergies, or participation in other clinical trials. Pregnant and/or lactating women will also be excluded.

#### 2.4.3. Participant Recruitment

The cardiologists will locate patients with CSAP and suggest that they participate in the study. The investigator will screen eligible patients according to the inclusion/exclusion criteria. Potential patients in local communities will be informed of the study through advertisements. Patients will be enrolled only if they meet the inclusion criteria and provide written informed consent.

### 2.5. Interventions

To ensure participant safety, we will comply with the European and Chinese guidelines for the management of patients with CSAP [5, 14]. All participants in the four groups will undergo the same basic treatment procedure. Additionally, following the guidelines, short-acting sublingual nitroglycerin therapy will be prescribed to all patients for the immediate relief of acute symptoms.

#### 2.5.1. Basic Treatment

Basic treatment includes health education and standard medications. Health education includes recommendations for lifestyle changes, such as increased exercise, reduced alcohol consumption, weight loss, and smoking cessation. Standard medications include 100 mg/d aspirin QD, 25 mg/d metoprolol BID, 5 mg/d ramipril QD, and 20 mg/d atorvastatin QN [5, 14].

#### 2.5.2. Plaster Production

In this study, the plasters for the AP therapy are divided into herbal medicine and placebo plasters. The herbal medicine plaster originates from a traditional Chinese decoction known as “guanxin suhe wan,” which was recorded in “The formulae of the Peaceful Benevolent Dispensary in the Song Dynasty.” This recipe includes styrax, borneol, frankincense, sandalwood, trichosanthes kirilowii Maxim, allium macrostemon Bunge, leeches, corydalis yanhusuo, and other ingredients. Clinical studies and animal experiments have identified a protective effect from this decoction on myocardial ischemia and hypoxia [15–19]. For the herbal medicine plaster, herbal medicine will be ground into fine powder and then mixed with honey to form a paste, which will be adhered to a special 5 × 5 cm sticking plaster with a 1.6 cm-diameter container in the middle. The placebo plaster will be made of buckwheat flour without the herbal medicines, but with the addition of honey and flavoring agents, the placebo plaster will have the same appearance, color, and smell as the herbal medicine plaster.

#### 2.5.3. Acupoint Selection

**Figure 2:**
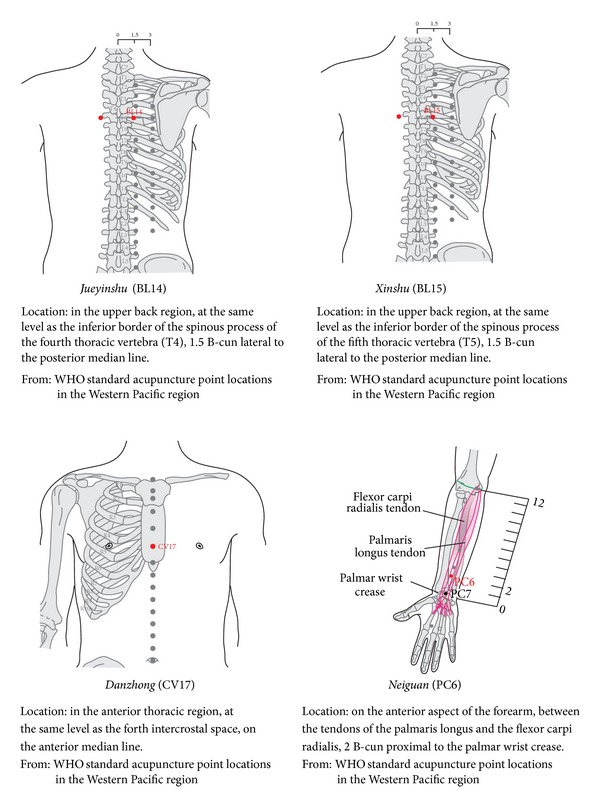
Diagram of acupoints location.

The acupoint selection regimen was developed by acupuncturists and cardiovascular specialists based on traditional acupuncture theories and our earlier literature review, which revealed that acupoints on the pericardium meridian were chiefly selected for angina [20]. The following acupoints will be used:* Jueyinshu *(BL14),* Xinshu* (BL15),* Danzhong* (RN17), and* Neiguan* (PC6); the names/codes and locations of all acupoints comply with WHO standards [21] (Figure 2). The nonacupoints are located in the same spinal segment but 2 cm away from the acupoints and outside the Chinese meridian system [22]. All acupoints and nonacupoints will be bilaterally used.

#### 2.5.4. Study Group

All groups will undergo the same basic treatment procedure, which continues from the baseline to the end of the follow up.

From the beginning of the study to the end of 4th week, the participants in the HAA group will receive the treatment of herbal medicine plaster on the acupoints and the participants in the PAA group will receive the treatment of placebo plaster on the acupoints; similarly, the participants in the HAN group will receive the treatment of herbal medicine plaster on the nonacupoints and the participants in the PAN group will receive the treatment of placebo plaster on the nonacupoints (Table 1). The operating procedure is to locate the acupoints, disinfect the skin, and attach the plaster on the acupoints. A total of 12 treatments will be performed during the 4 weeks, with one 6–8-hour treatment every other day for 6 days and one day of rest per week.

The plasters should be removed in advance if the local skin develops an allergic reaction to the medicine, which manifests as pruritus, redness, and erythra. When acute angina attack happens, the participant will receive an antianginal drug (nitroglycerin, nifedipine, or Suxiao Jiuxin pills [23]) according to the participant's previous treatment history and individual contraindications. For the majority of the participants, we will recommend short-acting sublingual nitroglycerin. Regardless of the medications, participants will be instructed to record drug information, including name, administration time, and dosage. The antianginal drugs will be provided for free. Nitroglycerin (Beijing Yimin Pharmaceutical Co., Ltd., Beijing, China), which is approved by the China Food and Drug Administration (CFDA, number H11021022), will be provided as a sublingual dose of 0.5 mg (one tablet); nifedipine tablets (CSPC Pharmaceutical Group Limited, Shijiazhuang, China, CFDA H13021315) will be provided as an oral dose of 10 mg (one tablet); finally, Suxiao Jiuxin pills (SX) (Zhongxin Pharma Tianjin number 6 Traditional Chinese Medicine Factory, Tianjin, China, CFDA Z12020025) will be provided as a sublingual dose of 5–10 pills. Other antianginal drugs will be prohibited. In the case of taking prohibited drugs, participants will be eliminated from the analysis due to protocol violation.

### 2.6. Investigator Training and Quality Control

All investigators involved in this study will receive theoretical and practical training courses to ensure that the operation is completely standardized. The training will include how to screen the eligible participants, use the random envelope, instruct participants to complete the diary cards, complete the case report form and assess efficacy and safety, and select acupoints and apply the plaster. After the training, all investigators will take a test. If they pass the test, they will obtain a certificate.

To guarantee the quality of the study, quality control is carried out every 3 months; that is, an inspection will be conducted by specially trained medical officers throughout the study.

### 2.7. Outcome Measurements

#### 2.7.1. Primary Outcome

The primary outcome is the change of the total frequency of self-reported angina attacks at the 4th week compared with week 0. This outcome will be determined from the participants' diaries.

#### 2.7.2. Secondary Outcomes

The secondary outcomes include the following: (1) intensity of angina pain on a VAS, which will be reported by the patient on a 100-point pain severity scale, where 0 indicates the absence of pain and 100 indicates the worst pain; (2) consumption of nitroglycerin or* Suxiao Jiuxin *pills (each participant can only receive one of these types to relieve acute symptoms); (3) the CCS angina class; (4) the SAQ score; (5) the SAS score and the SDS score; and (6) the change in the ST segment of the ECG.

The efficacy outcomes will be measured at weeks 0, 4, and 8. At baseline, the participants will undergo a physical examinations, outcome assessments, and regular tests. At 4th week and 8th week, the outcome assessments will be repeated. The regular tests include echocardiography; blood, urine, and stool tests; blood glucose and lipid tests; and liver and kidney function tests (Table 2).

#### 2.7.3. Assessment of Adverse Events

The risk of side effects of the AP therapy has been found to be low [10–12]. However, we will still examine any side effects of the treatment during the trial. All treatment-emergent AEs will be recorded during the treatment and the follow-up phase. Treatment-emergent AEs include itching, allergies, local infections, and other symptoms. AEs caused by antianginal drugs (basic treatment) will also be documented, and they include headaches, dizziness, nausea, flushing, and abdominal pain [4]. A serious adverse event (SAE) is defined as any AE that causes incapacity/disability, is life threatening or fatal, or requires hospitalization. Any AE/SAE will be documented in detail and reported. SAEs will be reported to the principal investigator and the ethics committee immediately. The principal investigator will decide whether the participant should withdraw from the study. The number of AEs will be calculated for each group.

### 2.8. Sample Size Calculation and Statistical Analysis

A previous study [24] found that the average weekly frequency of angina episodes of patients with CSAP was approximately 6.5 before treatment and the average reduction of weekly episodes was 3.7 in the AP group and 1.5 in the routine basic treatment group. Therefore, we predict that the weekly frequency of angina episodes of the HAA, PAA, HAN, and PAN groups will be 2, 4.5, 4.5, and 5 after treatment, respectively, with a combined standard deviation of 4. Assuming a 2-sided 0.05 significance level, a total of 172 patients (43 patients per arm) are needed to achieve a 90% probability of detecting improvement (G*Power software, Ver. 3.1.7). When considering a potential dropout risk of 15%, a total of 200 patients will be included in the study, with 50 patients in each group.

Statistical analyses will be performed by a statistician who is blinded to the treatments. Statistical nalysis Software (SAS, Ver. 9.1) will be used. The full analysis set (FAS) population is defined as all randomized patients who receive at least 1 treatment of the study regimen. The per-protocol (PP) population is defined as all patients in the intention-to-treat (ITT) population who are not major protocol violators. For the primary analysis, the PP population will be used. For the secondary analysis, the FAS and PP populations will be used. Demographic data at week 0 will be compared to measure the comparability among groups. For continuous variables, standard summary statistics will include mean, standard deviation, median, and maximum and minimum, and analysis of variance (ANOVA) with factor treatment will be performed. For categorical variables, the number and percentage will be tabulated, and chi-square tests will be used. The FAS population analysis will be based on the last observation carried forward (LOCF) for missing data.

## 3. Discussion

AP originates from long-term clinical practice by ancient Chinese doctors, who combined herbal medicine with acupoint stimulation on the basis of TCM theory. Through repeated summarization and innovation, a novel, external TCM therapy has been established [25]. The earlier studies for AP in the treatment of CSAP used self-controlled designs [10, 12, 26, 27], an active control compared with conventional drug therapy [24, 28, 29], a comparison of acupuncture with conventional drugs [30], a comparison of a transdermal therapeutic system of nitroglycerin with* Fufang Danshen Dripping* Pills [31], and a comparison of basic AP treatment with a nonacupoint regimen [11]. In these studies, the AP treatment groups were significantly superior to the control groups. However, biases resulted from poor-quality trial design, including a lack of baseline data, no placebo control groups, confused randomization methods, and conflicting concomitant medications. Therefore, we have designed a double-blind, randomized, placebo-controlled study to investigate the true efficacy of AP in the treatment of CSAP.

Is AP combined with acupoint stimulation and herbal medicine therapy superior to a single therapy, or is there a placebo effect? The answer cannot be determined from previous studies. We will observe clinical efficacy differences among different treatment regimens, including a combination of herbal medicine and acupoints (HAA group), acupoint stimulation only (PAA group), herbal medicine therapy only (HAN group), and a placebo treatment (PAN group).

In this trial, Suxiao Jiuxin pill is a compound developed by traditional Chinese medicine for cardiocerebral vascular diseases, which is listed in the national essential drug list of China [32]. The components of Suxiao jiuxin pill include Radix chuanxiong, and Borneolum syntheticum plus others [33]. It has been shown to relief angina pectoris and reduce the medication dosage and frequency of nitroglycerin [34, 35]. It was widely used to treat angiocardiopathy disease in China. With fewer side effects and the capability of rapid relief angina symptoms, it is accepted by many patients who suffer from angina pectoris and taken for prevention and treatment of angina pectoris. Suxiao Jiuxin pill is 40 mg per pill; a sublingual dose of 5–10 pills (200–400 mg) is recommended during attack of angina pectoris. In this study, we have chosen nonacupoint therapy as the control regimen, as investigators may not be blinded to the treatment, but they will remain blinded to the plasters. The participants will also be blinded to the treatments (acupoint selection and plasters) throughout the study.

## 4. Conclusion

This paper presents a design and protocol of AP for patients with CSAP. The study will evaluate the efficacy and safety of AP for this condition. The results of this trial will provide high-quality evidence for the use of AP in the treatment of CSAP.

## Figures and Tables

**Table 1:** Details of study groups.

Group	Acupoints	Interventions
HAA group	*Jueyinshu* (BL14), *Xinyu* (BL15) *Danzhong* (CV17), and *Neiguan* (PC6)	Herbal medicine application, acupoint, and basic treatment
PAA group		Placebo application, acupoint, and basic treatment
HAN group	Nonacupoints in the same spinal segment as those in the acupoints of HAA group but 2 cm away from genuine acupoints and outside the Chinese meridian system	Herbal medicine application, nonacupoint, and basic treatment
PAN group		Placebo application, nonacupoint, and basic treatment

**Table 2:** Study Schedule.

	Screening	Baseline (randomization)	Treatment	Follow up
Week	−4	0	4	8
Informed consent	×			
In/exclusion criteria		×		
Vital signs	×	×	×	
Medical history	×			
Concomitant disease/medication	×	×	×	×
Diary card distributed/review	×	×	×	×
Total frequency of angina attack		×	×	×
VAS scoring		×	×	×
Consumption of nitroglycerin or Suxiao Jiuxin dropping pills		×	×	×
CCS angina class		×	×	×
SAQ scoring		×	×	×
SAS and SDS scoring		×	×	×
ECG		×	×	×
Echocardiography		×		
Blood, urine, and stool tests		×		
Blood glucose and lipid tests		×		
Liver and renal function tests		×		
AEs		×	×	×
Reasons to withdrawal			×	×
Safety evaluation		×	×	
Compliance		×	×	×

CCS = the Canadian Cardiovascular Society; SAQ = Seattle Angina Questionnaire; SAS = self-rating anxiety scale; SDS = self-rating depression scale; VAS = visual analogue scale; ECG = electrocardiograph; and AEs = adverse events.
